# A randomised controlled trial of multimodal physiotherapy versus advice for recent onset, painful cervical radiculopathy – the PACeR trial protocol

**DOI:** 10.1186/s12891-019-2639-4

**Published:** 2019-06-01

**Authors:** Louise Keating, Caroline Treanor, Julie Sugrue, Dara Meldrum, Ciaran Bolger, Catherine Doody

**Affiliations:** 1 School of Physiotherapy, Royal College of Surgeons in Ireland (RCSI), Dublin, Ireland; 2 Department of Physiotherapy, Beaumont Hospital, Dublin, Ireland; 3 School of Medicine, Trinity College, Dublin, Ireland; 4 Department of Neurosurgery, Beaumont Hospital, Dublin, Ireland; 5 School of Public Health, Physiotherapy & Population Science, University College, Dublin, Ireland

**Keywords:** Cervical radiculopathy, Physiotherapy, Manual therapy, Exercise, Unloading tape, Pressure algometry

## Abstract

**Background:**

A research gap exists for optimal management of cervical radiculopathy in the first 12 weeks and short term natural history of the condition is somewhat unclear, although thought to be favourable. The primary aim of this assessor blinded, superiority, 2 parallel group randomised controlled trial is to investigate the effects of a 4 week physiotherapy programme (6–8 sessions) of manual therapy, exercise and upper limb neural unloading tape, compared to a control of weekly phone advice; on disability, pain and selected biopsychosocial measures, in acute and sub-acute cervical radiculopathy patients. A secondary aim is to identify whether any baseline variables, symptom duration or group allocation can predict outcome.

**Methods:**

Participants are recruited from GP referrals in an urban setting, from a neurosurgery non-urgent waiting list and from self-referral through Facebook advertising. Eligible participants (*n* = 64) are diagnosed with radiculopathy based on a clinical prediction rule and must have symptoms of unilateral, single level, radiculopathy for between 2 and 12 weeks, without having yet received physiotherapy. Random 1:1 group allocation (using variable block sizes), allocation concealment, blinded assessment and intention to treat analysis are being employed. Treatment is provided by clinical specialist physiotherapists in primary and secondary care settings. Outcomes are measured at baseline, 4 (primary endpoint) and 12 weeks. Participants’ report of pain, disability and their rating of recovery is also recorded by telephone interview at 6 months. Statistical analysis of between group differences will be performed with ANOVAs and MANOVAs, and multivariable regression analysis will be undertaken to explore predictor variables. Ethical approval for this study has been received from the Beaumont Hospital and Irish College of General Practitioners Research Ethics Committees. The trial is registered at ClinicalTrials.gov (NCT02449200).

**Discussion:**

An internal pilot study to test retention and recruitment strategies led to trial expansion and this is now a multi centre trial involving 5 clinical sites.

**Trial registration:**

NCT02449200. Registered 20/05/15.

## Background

Cervical radiculopathy (CR) has been defined by the North American Spine Society (NASS) as pain in a radicular pattern in one or both upper extremities related to compression and/or irritation of one or more cervical nerve roots, with signs and symptoms including varying degrees of sensory, motor, and reflex changes in addition to dysaesthesia and paraesthesia [1]. People with CR often experience high levels of pain and disability [2] and present to primary carers seeking diagnosis, reassurance and treatment, for a condition thought to have a favourable natural history of recovery over weeks and months [3, 4]. Ten years have passed since the Taskforce on Neck Pain highlighted the existence of a research gap for its optimal management [5], during the World Health Organisation’s (WHO) Bone and Joint Decade (2000–10); and although several clinical guidelines now exist for CR management [1, 3, 6, 7], they often rely heavily on consensus and have universally highlighted the paucity of high quality, randomised controlled trials.

The lack of quality trials is most evident in recent onset (12 weeks) CR, making evidence-based clinical decision-making a challenge for primary carers. Danish clinical guidelines recommend monitoring the individual patient’s clinical course to guide treatment decisions during this timeframe [6]. With a prevalence less than half that seen for lumbar radiculopathy [8], trial recruitment for recent onset (less than 3 months) CR is challenging.

Conservative treatment approaches described to date, have included pharmacology, advice to remain active, manual therapy, exercise, acupuncture, traction, collars and epidural injection [5, 6]. Manual therapy can include muscle energy techniques, high velocity manipulation or low velocity mobilisation of the cervical and/or thoracic spine, soft-tissue mobilisation and neural mobilisation techniques [9, 10]. Exercise in cohort studies and clinical trials has included mobility exercises, deep neck flexor and/or shoulder muscle endurance and strengthening [11, 12].

Not surprisingly, given the small evidence base for conservative management of CR, little is known about predictors of good clinical outcome. Cleland et al. [11] identified a four-variable model that detected participants who were most likely to demonstrate short-term, i.e. after 1 month, improvement with conservative treatment. This model included participants who were older than 54 years, whose dominant arm was not affected, whose symptoms were not aggravated by cervical flexion and who received multi-modal physiotherapy made up of manual therapy, cervical traction and deep neck flexor strengthening for at least half of their visits to the clinic. When all four variables were present, the positive likelihood ratio was 8.3 (95% CI = 1.9–63.9) [11].

The PACeR trial protocol is registered at ClinicalTrials.gov (NCT02449200) and is investigating the effectiveness of multimodal physiotherapy in comparison to an advice control, in recent onset CR.

### Aims

The trial’s primary aim is to investigate the effects of a multimodal physiotherapy (MP) programme on the independent primary outcome measures of disability, using the Neck Disability Index [13]; and pain, using the NPRS for both neck and arm pain at 4 weeks [14].

Secondary aims are;

To investigate the effects of the MP programme on selected biopsychosocial outcome measures at 4 and 12 weeks;

I. Patient reported outcome measures -
  ◦ Health-related quality of life using the SF-12 version 2 Health Survey [15].
  ◦ Mood using the Hospital Anxiety Depression Scale (HADS) [16].
  ◦ Fear avoidance using the Fear Avoidance Belief Questionnaire (FABQ) Neck [17, 18].
   ◦ Patient rating of recovery using the Global Rating of Change scale [19].

II. Clinical physical examination measures -
  ◦ Cervical range of motion measured with a CROM 3 device (Performance Attainment Associates, USA). Flexion, extension, bilateral side flexion and rotation are being measured.
  ◦ Pressure pain threshold (PPT) measured with pressure algometry [20], and the Upper Limb Neurodynamic Test (ULNT) 1 [21, 22] as measures of nerve mechanosensitivity.

- To identify whether any of the biopsychosocial outcome measures, as well as PainDETECT [23] at baseline, symptom duration and group allocation; can predict outcome (pain and disability) at 3 months.

### Hypothesis

A 4 week multimodal physiotherapy programme will lead to changes in self-reported disability and pain, compared to only advice to stay active, in patients with recent onset CR. The null hypothesis is that there will be no difference in pain and disability between the intervention group receiving a multimodal physiotherapy programme, and the control group receiving advice. Both groups can also continue to use medication throughout the trial, as prescribed by their GP.

## Methods

### Study design

The PACeR trial is an exploratory, two parallel group, assessor-blinded, multi-centre randomised controlled trial, with a primary endpoint of pain and disability at 4 weeks. This is a superiority trial to investigate a novel, complex physiotherapy intervention using allocation concealment, blocked randomisation and a 1:1 allocation ratio. The SPIRIT statement and CONSORT guidelines are guiding the reporting and conduct of the study [24, 25]. Ethical approval for this study has been granted by the medical research ethics committees of Beaumont Hospital (REC ref. 14/85), the Irish College of General Practitioners (ICGP) and the Royal College of Surgeons in Ireland (RCSI). Recruitment is via GP referral, self-referral through social media advertising, and initially, also from a waiting list of non-urgent referrals to a national neurosurgery centre. Five clinical centres (1 hospital and 4 private practices) are currently providing the trial’s interventions. Physiotherapy is provided by postgraduate master’s level or clinical specialist musculoskeletal physiotherapists.

### Eligibility criteria

Adults with single-level cervical radiculopathy of less than 12 weeks duration are eligible to participate. Inclusion & exclusion criteria are adapted from studies of similar cohorts [26, 27].

Inclusion criteria:

- Participants aged 18 years or older.
- Meet criteria for CR diagnosis on a clinical prediction rule (CPR) by demonstrating positive responses to at least 3 of the following clinical tests: Spurling’s Test, Upper Limb Neural Tissue Provocation Test 1 (ULNT 1), Cervical Distraction Test, and cervical rotation (less than 60°) on the symptomatic side [28].
- Complains of neck or periscapular pain in addition to radicular pain, paraesthesia or numbness in the upper limb; aggravated by neck posture or movement [29].
- Symptom duration must be greater than 2 weeks and less than 3 months.
- Mean of Numerical Pain Rating Scale (NPRS) scores for both neck and arm pain must be ≥3/10.
- Fluent in spoken & written English.

Exclusion criteria:

- Previous physiotherapy or manual treatment to cervical spine within past 6 months.
- Previous epidural injection since the onset of current symptoms.
- Prior surgery to the cervicothoracic spine or currently symptomatic upper limb.
- Current bilateral upper-limb symptoms.
- Myotomal paresis less than 4/5 on Medical Research Council (MRC) Scale in affected upper limb.
- Signs and symptoms suggestive of Cervical Spondylotic Myelopathy (CSM): bilateral paraesthesia, hyperreflexia, positive Babinski reflex and spasticity.
- Diagnosis of any generalised neurological disorder e.g. multiple sclerosis.
- Concurrent peripheral neuropathy affecting either upper limb e.g. carpal tunnel syndrome, thoracic outlet syndrome.
- Medical red flags suggestive of serious pathology such as neoplastic conditions, upper cervical ligamentous instability, vertebral artery insufficiency and inflammatory or
- systemic disease [30].
- Diagnosis of fibromyalgia.
- Psychiatric diagnosis in past 6 months.
- Ongoing litigation relating to cervical symptoms.

A two-step screening process is used to determine eligibility. A successful phone screen is followed by physical examination. Physical exam includes a neurological exam and manual exam of the cervical spine (C2-T2) to identify the symptomatic nerve root level.

If an MRI of the patient’s cervical spine has been undertaken, the report is reviewed for concordance after the initial assessment.

### Interventions

All participants are advised on CR’s natural history, positions of ease, to stay active and to take prescribed medication as appropriate.

**Fig. 1:**
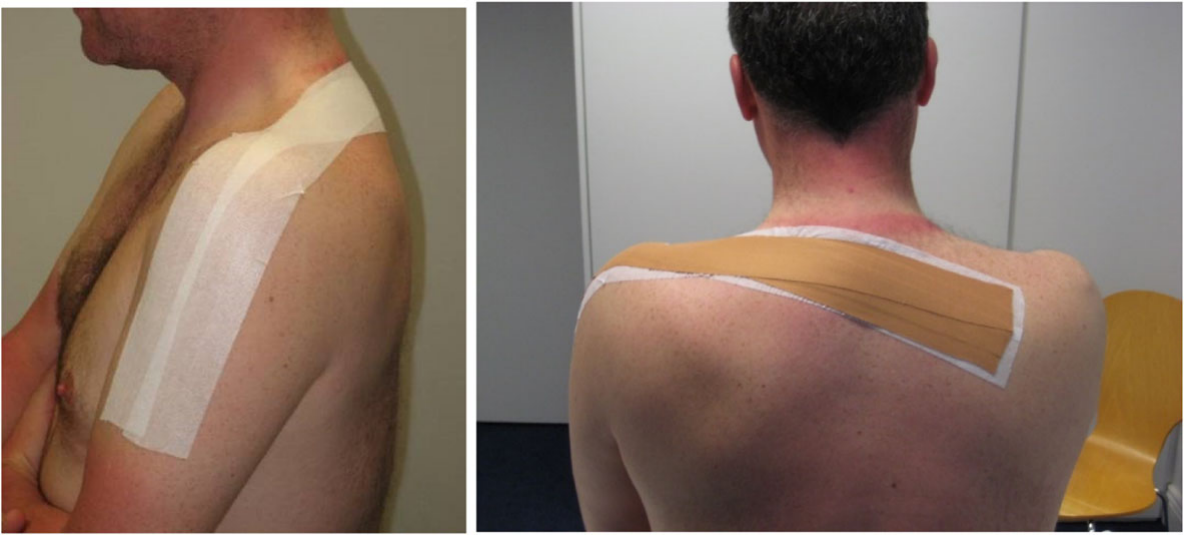
Neural unloading tape. Hypoallergenic Fixomull applied first as underwrap (left) with zinc oxide applied with tension, with arm supported below the elbow (right)

Participants allocated to the MP group receive 4 weeks (6–8 sessions) of non-provocative manual therapy, exercise and upper limb neural unloading tape (Fig. 1), as decided by the treating physiotherapist from a best-practice physiotherapy treatment manual. Previous RCTs investigating physiotherapy for nerve-related arm pain or cervical radiculopathy have involved 2 to 6 week programmes [27, 31, 32]. All participants receive lateral glide mobilisation [33] to the appropriate segmental level, applied using a treatment algorithm modified from Nee et al. [26]. Treating therapists are also permitted to use passive accessory or intervertebral mobilisations at this level, if the lateral glide does not yield clinical benefit. Additional joint mobilisations [34] to segmental levels above or below (C2 to T4) can also be applied, as deemed necessary by the treating physiotherapist e.g. to address segmental hypomobility.

All participants also receive exercise, including deep neck flexor (DNF) strengthening, mobility exercises and scapular muscle endurance exercise. The primary rationale for including exercise in this complex intervention is to achieve pain relief. Peripheral neuropathic pain and nociceptive pain mechanisms are known to co-exist in radiculopathy [35] and as such, it is to be expected that various exercises may be of benefit. Low level evidence already exists to support a variety of exercise types such as neck ROM, DNF training and scapular training, but primarily in the production of only short-term pain relief and to a lesser extent, functional improvement [31, 36], after 4 to 6 weeks of exercise. Given the painful and disabling nature of this condition, pain reduction even in the short-term is likely to be of value to the individual patient with CR. However, medium to long term improvement in pain and function, leading to improved social participation, should be a secondary focus of any exercise intervention in this cohort. A clear understanding of axioscapular muscle dysfunction has not yet been achieved for CR and so a pragmatic approach to exercise prescription has been adopted in this trial, with the intention of providing a bespoke programme to address actual deficits identified. Non-provocative, progressive endurance and strengthening for muscles of the neck and scapular region are also embedded into the programme and prescribed at the therapist’s discretion. As this intervention is of only 4 weeks’ duration, participants are advised to continue appropriate exercises until their 12 week assessment.

The third element to this complex intervention is upper limb neural unloading tape, adapted from McConnell [37]. Tape is applied in order to offset the upper limb load and provide relief of arm pain. Hypoallergenic 5 cm wide Fixomull and 3.8 cm zinc oxide tape is applied for 24–48 h. Treating therapists received 2-h training and agreed to follow the assessment and training manual. Recording of treatment sessions was done using standardised report forms.

Control group participants are phoned by a physiotherapist on a weekly basis for 4 weeks. They provide an update on their symptom profile and receive advice.

All participants are asked to forego any other physiotherapy external to the trial prior to the 12 week time point. To optimise retention, control group participants are offered treatment at 12 weeks.

### Outcome measures

Table 1 outlines the SPIRIT schedule, which summarises the schedule of enrolment, interventions, and assessments.

**Table 1:**
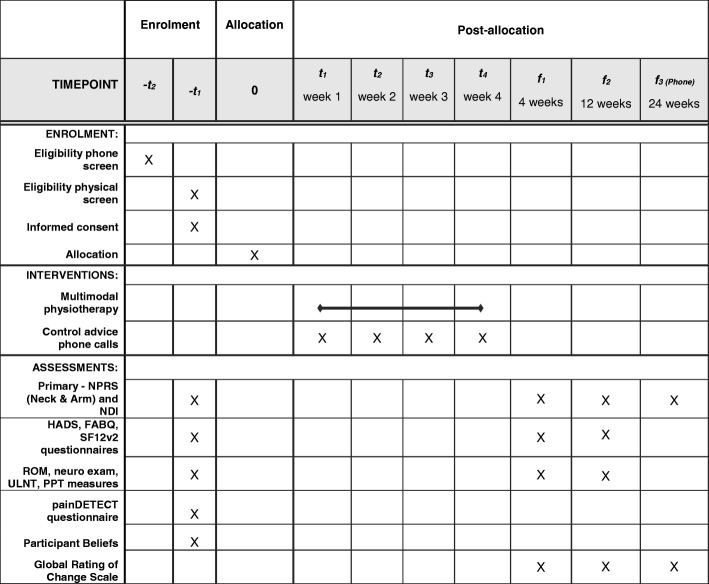
PACeR trial schedule of enrolment, interventions, and assessments adapted from original table© SPIRIT Group [48]

A CPR with established diagnostic accuracy is used to confirm the presence of CR [28]. This involves 4 physical tests, which include measurement of cervical rotation and Upper Limb Neurodynamic Test (ULNT) 1. Cervical rotation range is measured with a CROM 3 device (Performance Attainment Associates, USA) made up of 3 cervical inclinometers. Reliability (ICC = 0.89–0.90) and construct validity of the CROM have been demonstrated [38]. ULNT 1 is a validated pain provocation test for nerve tissue [21, 22], which has also demonstrated moderate reliability (kappa = 0.45) [39].

Participants undergo the following outcome measures at baseline, 4 and 12 weeks:

The *Neck Disability Index* is a ten-item self-reported questionnaire that assesses pain and associated disability [13]. The NDI is valid and reliable for use in cervical radiculopathy [40]. The Taskforce on Neck Pain considered it to be the most responsive self-assessment questionnaire available [41] due to its ability to discriminate between clinical improvement and deterioration in neck pain [42].

The *Numerical Pain Rating Scale,* a reliable and responsive [43] generic measure of pain [14] is being used to measure both neck and arm pain.

Cervical *range of motion* measured with a CROM 3 device (Performance Attainment Associates, USA). Flexion, extension, side flexion and rotation, along with symptom response, is being measured.

The *SF-12 Health Survey* questionnaire will be used as a measure of quality of life (QoL) and is a practical, reliable, and valid measure of physical and mental health [44, 45].

The *Hospital Anxiety and Depression Scale (HADS)* and the *Fear Avoidance Belief Questionnaire (FABQ)* are used as psychosocial measures. The HADS [16] was developed as a tool to identify anxiety and depression in patients attending non-psychiatric hospital outpatient clinics and has been validated [46]. The FABQ was initially established to measure how patients’ beliefs about physical activity and work affected their low back pain [17]. FABQ has also been shown to predict prolonged disability in neck pain patients, making it appropriate to use with this cohort of neck pain patients [18].

*Pressure Pain Threshold* (PPT) is measured using pressure algometry and provides a measure of mechanosensitivity of the nervous system [20]. Good inter and intra-rater reliability of PPT has been established in a neck pain cohort [20]. A similar profile of altered mechanosensitivity previously found in Whiplash Associated Disorder (WAD) patients, has also been identified in patients with chronic cervical radiculopathy [47]. PPT is measured using the SENSEBox algometer (Somedic, Sweden) with a 1cm^2^ head, applied at a rate of 30 kPa/sec, at standardised sites (dermatomal key points and maximal pain area) bilaterally, and on the right anterior tibia. The mean of 3 measurements at each site, recorded at 10 s intervals, and a random site order is applied.

Intra-rater reliability of the PI will be verified for the ULNT 1 and PPT measurements by conducting repeat tests for 10 participants. Intraclass Correlation Coefficients (ICC) will be calculated using a one-way random effect model (for consistency agreement).

*PainDETECT,* a self-report questionnaire to detect neuropathic pain components, originally designed for use in low back pain [23] is also being used at baseline. This questionnaire has excellent test-retest reliability (ICC = 0.93), good internal consistency (Cronbach’s alpha > 0.83) and high sensitivity, specificity and positive predictive value (> 80%) [23].

Participants’ overall rating of recovery is assessed using the *Global Rating of Change scale* (GROC) [19] at 4 and 12 week follow-up.

A final phone follow-up at 6 months will capture GROC, NPRS, NDI and any additional treatment received.

In addition to these outcome measures, baseline demographics including co-morbidities, educational level, smoking status, occupation and work status will also be captured; with change in work status noted at subsequent time points.

Medication use for both groups is recorded weekly over the first month by physiotherapists and by the PI at follow up assessments. Adherence to home exercise prescription is also recorded on a weekly basis in the MP group and again at the 12 week assessment. The number of treatment sessions attended by participants will also serve as an additional measure of treatment adherence.

### Randomisation

The allocation sequence has been generated by an independent academic colleague using a computer-generated list of random numbers and randomly varied block sizes of 4 and 6 from www.randomization.com. The allocation sequence is concealed from the PI, who enrols and consents participants. The PI informs the randomiser when a new participant has been enrolled and once randomised, the randomiser directly informs the relevant physiotherapist, who informs the participant of their group allocation by phone. Participants are randomly assigned using a 1:1 group allocation ratio. The PI is blinded to group allocation until the 12 week assessment. Given the nature of the interventions, it is not possible to blind the participants or therapists involved in providing either intervention.

### Sample size estimation

The NDI and the NPRS are the independent primary outcome measures and sample size estimates were carried out for both outcome measures. An MCID for the NDI of 7 has been determined for cervical radiculopathy [48]. Using a reported NDI SD of 9.2 [48] for this patient group, with a two-sided 5% significance level and a power of 80%, a sample size of 29 participants per group is necessary. An MCID for the NPRS of 2 has been determined for mechanical neck pain, including cervical radiculopathy [43] and a SD of 1.85 [48]. Using these figures, with a two-sided 5% significance level and a power of 80%, a sample size of 15 per group was calculated. An online sample size calculator [49] for comparing means of 2 independent groups, that utilises reference tables [50] was used. The larger sample size per group calculated using the NDI was chosen and an additional 10% was added to account for anticipated dropout. In total, a sample size of 64 participants will be recruited.

### Data analysis plan

Baseline demographic characteristics and all outcome measures will be described and analysed using SPSS (IBM, version 24) or Stata statistical software (Statacorp LLC, Release 15). Descriptive characteristics will be presented for both groups following the CONSORT Statement. Data will be checked for normality using the Shapiro-Wilk test. Marginal effects will be used to calculate effect sizes for specified values for baseline differences. Results will be reported as mean differences between the groups and their 95% confidence intervals. Poisson regression will be used where data are non-parametric (Bland and Altman, 2011).

Statistical analysis will include two-way (treatment x time) ANOVAs and MANOVAs for between and within-group differences and interactions. Intention to treat analysis will be the primary approach employed. A per protocol analysis will also be undertaken and results compared to assess the impact of data that is not missing at random (NMAR) (Armijo-Olivo et al., 2009).

Secondary analysis will explore if any of the baseline outcome measures, symptom duration and group allocation are predictors of outcome (pain and disability) at 3 months. Appropriate multivariable regression analysis will be performed (linear or logistic).

## Discussion

An internal pilot feasibility study of 10% sample has explored the initial recruitment strategy and retention rates of the study, in alignment with MRC Guidelines (MRC, 2008). Retention strategies proved acceptable but recruitment strategies were insufficient, leading to geographical trial expansion and the addition of self-referral through social media advertising. This trial of non-provocative multimodal physiotherapy for recent onset CR, will elucidate whether physiotherapy actively improves the pain and disability associated with the condition, when compared with advice only.

## Data Availability

Not applicable.

## Abbreviations

CPR: Clinical prediction rule
CR: Cervical Radiculopathy
CROM: Cervical range of motion
CSM: Cervical Spondylotic Myelopathy
FABQ: Fear Avoidance Belief Questionnaire
GROC: Global Rating of Change Scale
HADS: Hospital Anxiety and Depression Scale
ICC: Intraclass correlation coefficient
MCID: Minimal clinically important difference
MP: Multimodal physiotherapy
MRC: Medical Research Council
NASS: North American Spine Society
NDI: Neck Disability Index
NMAR: Not missing at random
NPRS: Numerical Pain Rating Scale
PI: Principal investigator
PPT: Pressure Pain Threshold
RCSI: Royal College of Surgeons in Ireland
RCT: Randomised controlled trial
ULNT: Upper Limb Neurodynamic Test
WHO: World Health Organisation
